# Mobile Messaging Support Versus Usual Care for People With Type 2 Diabetes on Glycemic Control: Protocol for a Multicenter Randomized Controlled Trial

**DOI:** 10.2196/12377

**Published:** 2019-05-30

**Authors:** Andrew Farmer, Kirsty Bobrow, Natalie Leon, Nicola Williams, Enita Phiri, Hazel Namadingo, Sara Cooper, John Prince, Amelia Crampin, Donela Besada, Emmanuelle Daviaud, Ly-Mee Yu, Jonathan Ngoma, David Springer, Bruno Pauly, Shane Norris, Lionel Tarassenko, Moffat Nyirenda, Naomi Levitt

**Affiliations:** 1 Nuffield Department of Primary Care Health Sciences University of Oxford Oxford United Kingdom; 2 Chronic Disease Initiative for Africa University of Cape Town Cape Town South Africa; 3 South African Medical Research Council Cape Town South Africa; 4 Malawi Epidemiology and Intervention Research Unit Lilongwe Malawi; 5 Institute of Biomedical Engineering Oxford United Kingdom; 6 Malawi Epidemiology and Intervention Research Unit London School of Hygiene and Tropical Medicine London United Kingdom; 7 Kamuzu Central Hospital Lilongwe Malawi; 8 The Boston Consulting Group London United Kingdom; 9 Department of Diabetes and Endocrinology Chris Hani Baragwanath Academic Hospital Johanesburg South Africa; 10 South African Medical Research Council Developmental Pathways for Health Research Unit University of Witwatersrand Johannesburg South Africa

**Keywords:** randomized controlled trial, diabetes mellitus, type 2 diabetes, mobile health, treatment adherence

## Abstract

**Background:**

Health outcomes for people treated for type 2 diabetes could be substantially improved in sub-Saharan Africa. Failure to take medicine regularly to treat diabetes has been identified as a major problem. Resources to identify and support patients who are not making the best use of medicine in low- and middle-income settings are scarce. Mobile phones are widely available in these settings, including among people with diabetes; linked technologies, such as short message service (SMS) text messaging, have shown promise in delivering low-cost interventions efficiently. However, evidence showing that these interventions will work when carried out at a larger scale and measuring the extent to which they will improve health outcomes when added to usual care is limited.

**Objective:**

The objective of this trial is to test the effectiveness of sending brief, automated SMS text messages for improving health outcomes and medication adherence in patients with type 2 diabetes compared to an active control.

**Methods:**

We will carry out a randomized trial recruiting from clinics in two contrasting settings in sub-Saharan Africa: Cape Town, South Africa, and Lilongwe, Malawi. Intervention messages will advise people about the benefits of their diabetes treatment and offer motivation and encouragement around lifestyle and use of medication. We allocated patients, using randomization with a minimization algorithm, to receive either three to four intervention messages per week or non-health-related messages every 6 weeks. We will follow up with participants for 12 months, measuring important risk factors for poor health outcomes and complications in diabetes. This will enable us to estimate potential health benefits, including the primary outcome of hemoglobin A_1c_ (HbA_1c_) levels as a marker for long-term blood glucose control and a secondary outcome of blood pressure control. We will record the costs of performing these activities and estimate cost-effectiveness. We will also use process evaluation to capture the collection of medication and assess the reception of the intervention by participants and health care workers.

**Results:**

Recruitment to the trial began in September 2016 and follow-up of participants was completed in October 2018. Data collection from electronic health records and other routinely collected sources is continuing. The database lock is anticipated in June 2019, followed by analysis and disclosing of group allocation.

**Conclusions:**

The knowledge gained from this study will have wide applications and advance the evidence base for effectiveness of mobile phone-based, brief text messaging on clinical outcomes and in large-scale, operational settings. It will provide evidence for cost-effectiveness and acceptability that will further inform policy development and decision making. We will work with a wide network that includes patients, clinicians, academics, industry, and policy makers to help us identify opportunities for informing people about the work and raise awareness of what is being developed and studied.

**Trial Registration:**

ISRCTN Registry ISRCTN70768808; http://www.isrctn.com/ISRCTN70768808 (Archived by WebCite at http://www.webcitation.org/786316Zqk)

**International Registered Report Identifier (IRRID):**

DERR1-10.2196/12377

## Introduction

### The Global Health Importance of Diabetes Mellitus

Diabetes mellitus, specifically type 2 diabetes, is a major burden to individuals and health care systems globally, including in low-resource settings. Estimates of the prevalence of diabetes in Southern Africa vary. A 2009 survey across South Africa placed the incidence at 9% [1], but a survey in Cape Town identified a rising prevalence with an age-adjusted prevalence of 13.1% [2] in black Africans. The prevalence of diabetes is also rising in Malawi with an incidence of 6% [3]. As the prevalence of diabetes is rising, it is likely that the impact of the associated premature mortality and morbidity through, for example, visual impairment, renal dysfunction, neuropathy, and cardiovascular disease will increase. However, failure to take medicine as prescribed, often referred to as nonadherence, can result in a failure to deliver the benefits of effective medical treatments into better outcomes for individual patients. For example, there is an association between better adherence to treatment and better control and fewer complications for people with type 2 diabetes [4,5].

### The Need for Further Research and Interventions to Improve Regular and Sustained Use of Medication

Reasons for not collecting or taking medications as intended are well documented and include psychological factors, lack of social support, low levels of health literacy, and interactions with the health care system that do not support self-management [6]. Medication adherence in sub-Saharan Africa is estimated at around 64% [7]. Better understanding of treatments and helping people deal with day-to-day challenges can improve the collection and taking of medicines [8]. Interventions delivered by short message service (SMS) text messaging have been effective in increasing adherence to antiretroviral therapy and other conditions [9,10]. More research is needed to develop and test better ways to leverage widely used new technologies to help people to improve their use of medicine.

### Mobile Health-Based Support for People With Long-Term Conditions

In a primary care setting in the Western Cape [11], we developed a low-cost system of registering patients and regularly sending health messages via SMS text messages. A randomized trial of the intervention for people with high blood pressure—the SMS-Text Adherence Support-Blood Pressure (StAR-BP) trial [12]—showed better adherence and improved blood pressure control for people receiving the SMS text messages compared to those who received active control messages (ie, usual care supplemented by noninformational text messaging). Our system of sending text messages complies with the principles of the information and communications technology policy frameworks now being established in sub-Saharan countries, including the South African mHealth Strategy [13]. For example, our system aims to be integrated into routine care and simple in design. As part of our work to understand the broader applicability of the intervention for people with other chronic diseases, we are now exploring its use for people with type 2 diabetes.

Our own recent focus group work and interviews alongside the StAR-BP trial [14] confirm the importance of offering evidence-based information to help with self-management of long-term conditions. For example, lack of knowledge and mixed feelings toward regular use of medicine to treat chronic conditions, as well as difficulty in remembering to take them, can lead to missing clinic appointments for medicine collection and missing out on taking the tablets. Participants in the StAR-BP trial told us how helpful the system was in reminding them to collect and use their medication. They also told us it encouraged them to make greater efforts to maintain their general health [14].

A systematic review of SMS text messaging in supporting medication use in type 2 diabetes suggested significant benefits, but there was substantial heterogeneity. A recent study in New Zealand that included people with type 1 diabetes and type 2 diabetes with insulin treatment also suggested a benefit in glycemic control. Other work has been carried out to evaluate diabetes-focused SMS systems in low-resource settings where very low rates of medication use offer potential for substantial benefit. In a two-center comparison study in Senegal, text messages were sent to people with diabetes over 3 months. A 6-month randomized trial in Bangladesh consisting of 256 patients identified a small but significant fall in hemoglobin A_1c_ (HbA_1c_) [15]. However, a longer study duration, the use of structured message development, and studies in sub-Saharan Africa are needed to reduce uncertainty.

We therefore adapted the message content for the StAR-BP system to be appropriate for people with type 2 diabetes in two sub-Saharan settings. Content was based on development work to identify messages that could provide support through behavior-change techniques as proposed by the Capability, Opportunity, Motivation-Behavior (COM-B) framework, which focuses on capability, opportunity, and motivation factors for changing behavior [16-18]. We followed recommended guidelines on SMS text message development, including testing and adapting for local applicability as well as usefulness and field-testing the intervention. Feedback was elicited from a range of stakeholders, including diabetes patients, diabetes health care staff, and experts, concerning promotion of a healthy lifestyle, diet, and exercise in order to understand relevance, usefulness, and acceptability of message content. The system offers a new approach to improving access to care through providing support to patients with diabetes alongside usual care.

In doing this development work, we followed the Medical Research Council framework for developing and evaluating complex interventions [16]. We have also used theory- and evidence-informed behavior-change constructs to guide the content and delivery mechanisms of the SMS text message intervention in this setting [19]. The explicit use of this approach for diabetes messaging in this context is novel [20]. We planned to test effectiveness and cost-effectiveness of the system in improving glycemic control using an individually randomized controlled design with an embedded process evaluation.

## Methods

### Trial Aims and Objectives

The overall aim of the SMS-Text Adherence Support for Type 2 Diabetes (StAR2D) trial is to test the effectiveness of sending SMS text messages in improving health outcomes and medication adherence in patients with type 2 diabetes compared to an active control. A secondary aim is to examine the incremental cost and cost-effectiveness of the intervention. A process evaluation using qualitative research methods will be carried out alongside the trial to investigate participant responses and acceptability.

### Ethical Issues

The University of Oxford Tropical Research Ethics Committee (OXTREC) approved the research protocol (reference number 22-15). Additionally, the University of Cape Town Human Research Ethics Committee (UCT HREC) (reference number 126/2015) and the Malawi National Health Services Research Committee (NHSRC) (reference number 15/7/1425) also approved the protocol. The sponsor of this study—University of Oxford—has put in place insurance in the event that any participant suffers harm as a result of their involvement in the research. This protocol refers to version 2.0 of the StAR2D trial protocol, dated September 16, 2016, that was submitted following formative work but before recruitment to the trial began.

### Trial Design

**Figure 1:**
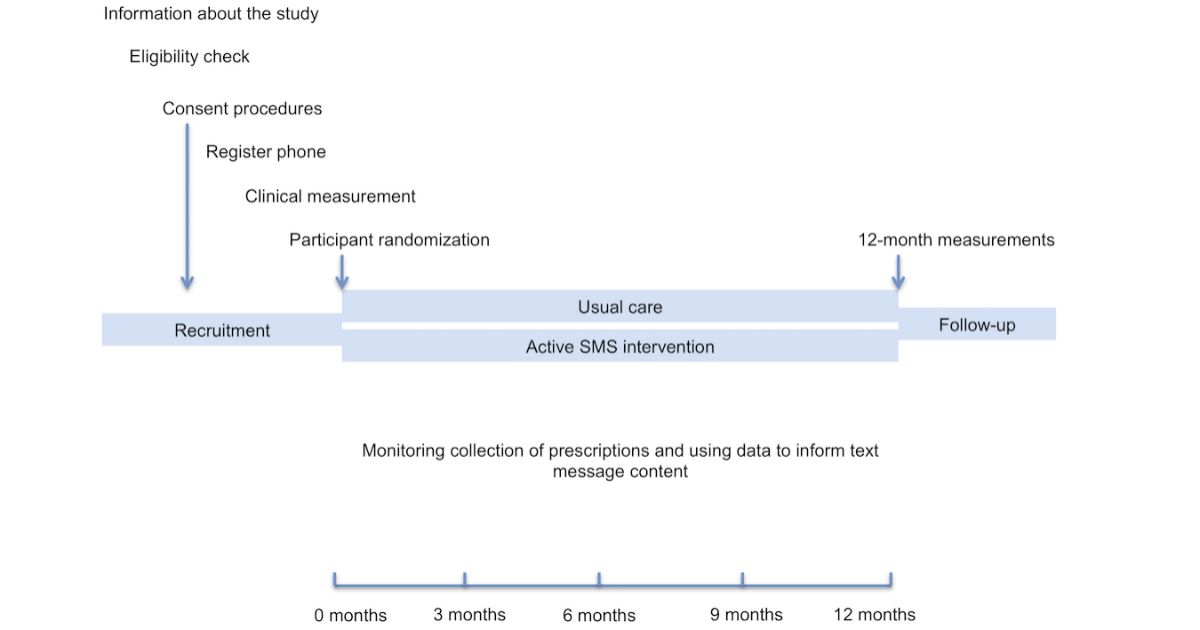
Trial flowchart. SMS: short message service.

The trial is a 12-month, multicenter, two-parallel-arm, individually randomized controlled trial. A flowchart showing the sequence of recruitment, assessment, and intervention is shown in Figure 1. A checklist of trial procedures is given in Table 1.

**Table 1:** Visits and procedures to be performed during the trial.

Procedures	Visits			
	Eligibility check	Baseline	Follow-up session with routine data and nonscheduled research contacts	12-month visit
Eligibility assessment Duration of diabetes	X			
Demographics: age, gender, language preference, and work status		X		
Medical history: comorbid conditions and duration of diabetes		X		
Current medication, including current oral glucose medication, doses, use of insulin, and other medications	X	X		X
Tobacco use		X		X
Changes in medication			X	X
Physical examination: height and weight		X		X
Hemoglobin A_1c_ measurement		X		X
Total and HDL^a^ cholesterol		X		X
Blood pressure measurement		X		X
Medication pickup		X	X	X
Adherence score		X		X
Purchase of drugs during stock-out (ie, out of stock at clinic)				X
EuroQol 5-Dimension 3-Level		X		X
Satisfaction with care		X		X
Self-reported measures of eating and physical activity		X		
Nonscheduled research contact			X	
Adverse event assessment			X	X
Failure to receive message			X	

^a^HDL: high-density lipoprotein.

### Setting

Participants were recruited from publicly funded outpatient facilities that treat people with type 2 diabetes. We identified sites in Cape Town, South Africa, and Lilongwe, Malawi, serving patients living in low- and middle-income settings. Both are urban sites with a high burden of type 2 diabetes. In Cape Town, recruitment was from two clinics serving townships to the north of the city, and Lilongwe is the capital city of Malawi. A high proportion of the population live in low-income informal settlements where one clinic was involved in recruitment. In both settings, patients received health care and medication from a locally appropriate health care facility. Care is provided free of charge and a limited range of essential medicines are available at no cost to patients to reduce blood glucose and blood pressure.

### Participant Eligibility, Recruitment, and Screening

Eligible patients were those with type 2 diabetes, aged 18 years or greater, and taking an oral glucose-lowering medication. Other inclusion criteria included the following: ability to communicate in one of the predominant official languages spoken in the Western Cape province in South Africa (English, Afrikaans, or isiXhosa) and in Malawi (English or Chichewa language); access to a mobile phone, where shared access is allowed with permission of the phone owner; ability to use, or be helped to use, the SMS text messaging feature on a mobile phone, including knowing that a text message had been received and reading it; and current and planned future residence in the communities served by the participating clinics. The following patients were ineligible for recruitment: patients who have been admitted to hospital for hyperglycemia or hypoglycemia within the previous 3 months; patients who are pregnant or within 3 months postpartum by self-report or with plans to become pregnant in the next 12 months; patients with a terminal medical condition; patients with another member of the household already recruited to the trial; or patients who participated in formative work for the intervention development.

Patients attending the clinic for their regular diabetes care were provided with information about the trial at the regular education sessions, on posters, and in leaflets. Clinic patients who attended for routine diabetes care or to pick up diabetes medicine and who have received information about the trial were asked if they were interested in joining the trial.

Potential trial participants received verbal and written information about the trial in their preferred language and had the opportunity to speak to trained study research staff and ask them questions. If they agreed to participate, they provided verbal assent to screening procedures. If eligible, they then provided written consent for enrolment and to enter the trial. Copies of the consent forms were given to participants and randomization was not carried out until confirmation that a “welcome” text message had been received.

Data were transmitted using a low-cost, advanced-feature mobile phone (ie, smartphone) acting as the trial data collection system. A secure implementation of Sana (MIT), which ran on the mobile phone, was linked to a secure server running Open Medical Records System (OpenMRS) [21] using secure information exchange protocols. This allowed errors in data entry to be checked and enabled immediate upload of data to the trial server.

### Randomization

We used a remote Web-based randomization program, Sortition (Oxford), minimizing for time since diagnosis, age, sex, and trial site. Adults with type 2 diabetes were allocated in a 1:1 ratio to receive automated text message support or usual care supplemented by active control. Allocations were directly uploaded into the OpenMRS database to avoid creation of locally held records. Randomization was carried out remotely and independently of the clinic and local research staff. No arrangements for unblinding during the trial were made.

### Trial Outcomes and Other Measures

The primary outcome of the trial is the change in HbA_1c_ from baseline to 1 year. Secondary clinical outcomes are the proportion of patients collecting 80% or more of their agreed-upon, diabetes-related medication derived from routine clinic data [22]; change in systolic blood pressure; change in lipids; a combined measure of cardiovascular risk based on HbA_1c_, lipids, and systolic blood pressure [23]; and the proportion of the participants reaching treatment goals (ie, HbA_1c_ <8% and systolic blood pressure <140 mmHg).

The EuroQol 5-Dimension 3-Level (EQ-5D-3L) instrument [24] and a locally adapted questionnaire to establish satisfaction with treatment and delivery of treatment [25,26] were used and available in all of the study languages. In addition, self-reported medication-taking was recorded [27]. Basic demographic data collected included age, sex, language preference, and work status. Anthropometric measures were collected, including measurement of height and weight using standard procedures and self-reports of eating and physical activity, with a 7-day recall.

Measurement and collection of data were carried out by a team of research assistants trained and supervised to ensure consistency between and within study sites with standard operating procedures for clinical measurement.

### Follow-Up Assessment

Annual follow-up is integrated with routine health care review. At 12 months, a reminder text will be sent to invite participants to attend their annual health care review and a final trial assessment. Participants who do not attend the 12-month follow-up clinic appointment will be followed up using the mobile phone and other contact details; if the cause of nonattendance is hospital admission or death, then hospital records will be obtained.

### Intervention

Trial participants allocated to the intervention group received specifically designed text messages, including motivational and educational messages. They also received prompts (ie, reminders) about medication collection with timing personalized by the information collected about all participants at the baseline visit, from the clinic and pharmacy attendance. Messages were sent three to four times a week for a period of 1 year. A message was sent giving options to change the delivery time or language of messages. Messages were randomly selected from a library, using rules that ensured individual messages were not repeated. Messages were also personalized by information given about smoking and use of alcohol. Full details of the intervention are given in Multimedia Appendix 1. The intervention included information intended to encourage people to take their medicine regularly as prescribed. The intervention also prompted participants when an anticipated attendance has not occurred and informed them when an out-of-stock medicine was received in the pharmacy and patients needed to return to obtain it. The intervention is summarized in the Template for Intervention Description and Replication (TIDieR) statement uploaded as Multimedia Appendix 2.

Trial participants allocated to the usual care group received an active control protocol: only noninformational text messages were sent (eg, messages thanking the participant for taking part in the study and a message on their birthday), alongside usual care every 6 weeks. At both study sites, usual care consisted of attendance to collect medication supplies at 2-monthly intervals with review appointments where clinically indicated. Health material on type 2 diabetes were available at all sites, which included information about the importance of taking medicine regularly, alongside other health information. Attendance at all appointments by trial participants was tracked through routinely implemented electronic and manual registers. Messages were stopped at participant request.

### Sample Size Estimation

We consider a minimum reduction in HbA_1c_, from baseline to 12 months, of 0.5% in this population clinically important and feasible. A total of 814 participants (407 per group) across all sites would be required to show a 0.5% reduction—assuming, conservatively, a standard deviation of 2.2%—in this population with 5%, two-sided significance level and power of 90% using PASS software version 12 (NCSS). We have increased the number to account for potential clustering between sites and loss to follow-up of up to 20% to a total of 1066 (ie, 533 per group). We will also have 90% power to detect a 10%-point increase in adherence rate in the intervention group (ie, from 50% to 60%), including adjustment for clustering effects. We plan to recruit half of the participants in each setting.

### Analyses

The primary analysis will be carried out on the basis of intention-to-treat (ITT) analysis. We will endeavor to obtain full follow-up data on every participant to allow full ITT analysis, but we expect missing data due to withdrawal, loss to follow-up, or failure to attend clinic visits. The results from the trial will be prepared as comparative summary statistics with 95% confidence intervals. All the tests will be done at a 5%, two-sided significance level. The study results will be reported in accordance with the Consolidated Standards of Reporting Trials (CONSORT) 2010 statement. A full, detailed, statistical analysis plan will be prepared and finalized before participant follow-up is completed.

A linear regression model will be used to compare the primary outcome (ie, change in HbA_1c_ from baseline to 1 year) between groups, adjusting for baseline HbA_1c_ and minimizing variables. Similar methods will be used to analyze blood pressure data and other continuous outcomes. The proportion of people with more than 80% proportion of days covered with medication will be compared using adjusted logistic regression. We will carry out a prespecified secondary analysis to compare outcomes in individuals with uncontrolled diabetes (ie, HbA_1c_ >8%) at baseline.

Missing data will be reported with reasons given where available and the missing data pattern and mechanism will be explored. We will also carry out various sensitivity analyses using alternative imputation methods to examine the robustness of the results. Finally, we will use the regression method to determine the factors influencing the impact of the intervention.

We will carry out subgroup analyses of the primary outcome and adherence outcomes for the following subgroups: age (<55 years or ≥55 years); site (Cape Town or Lilongwe); sex (male or female); number of years with type 2 diabetes (<7 years or ≥7 years); presence of one or more comorbidity (none, one, or more); diabetes control at baseline (HbA_1c_ ≤8% or >8%); and self-reported adherence rating score at baseline (25 or <25).

### Economic Analysis

The costing study will be based on data collected during the trial and by using the trial findings. The costing study will provide a descriptive account of the different cost components annualized to take into account development and capital costs in the form of a percentage increase in costs; the cost-effectiveness study will model the system in terms of cost per disability-adjusted life year (DALY) averted.

To assess the added cost per patient-year, the one-off design costs will be excluded. Setup costs will be annualized over a period of 10 years to reflect their potential for use in scale-up and other applications and added to 1-year delivery costs. Capital costs will be calculated by the replacement value of each item and the estimated useful life and then annualized with a discount rate of 3%. Costs will be adjusted for time using the consumer price index. We will carry out a descriptive analysis of the observed costs using standard methodologies. To assess additional costs as a proportion of nonintervention delivery costs per patient-year, total costs per treatment per patient-year will be calculated.

Economic costs will include provider and patient costs. Facility-level expenditure will include clinical staff, supplies, and overhead (ie, capital, support staff, utilities, administration, and management). Overhead will be calculated based on the level of diabetes clinical activity as a share of the facility activity. Number of visits, laboratory tests, and medication will be collected from the project database. Besides facility costs, costs associated with the SMS text messaging will be included. One-off costs of the initial design of the system (ie, equipment capital costs, staff, and supplies) will be identified. Research costs, apart from that component of the formative research that would be required if the intervention were rolled out to new districts, will be excluded. Data sources will include interviews with staff, facilities’ financial records (ie, staff packages and unit costs of drugs, laboratory tests and other supplies, and overheads), and financial records of the SMS organization. Patient costs will include recording the time spent in clinic and asking about time spent travelling and costs of transport to the clinic. These questions will only be asked once. The study project manager and local coordinators will be interviewed to validate the information.

The costing outcome will be the cost per patient-year and the cost-effectiveness outcome will be incremental cost per DALY averted [28,29].

### Process Evaluation

The process evaluation is aimed at understanding implementation of the intervention and contextual factors that may explain the effects and applicability of the intervention. In particular, we will explore the reach of and patient responses to the intervention, including acceptability, in order to enhance our understanding of why and how the intervention worked or not and how it can be optimized in the future. Process measures on the intervention reach and fidelity will be collected from the SMS text message system where numbers of participants and numbers of text messages sent and received are stored. At each site, sources of data will be used to measure activity in the clinic (eg, numbers attending the clinic) and the pharmacy. We will also use these measures during the period of the study to identify where problems might develop and to explore differences between sites.

To explore how the intervention was received and responded to, we will use semistructured interviews and focus groups with purposive and convenience sampling of stakeholders, mainly patient participants, but also clinic staff and representatives from the relevant department of health involved with the study. We will also conduct document reviews relating to the implementation of the trial. Purposive sampling aims to explore variation in response by variables, including age, gender, and language group of patient participants. Patient participant interviews will explore their experiences and views of receiving SMS text messages; their responses (ie, thoughts, feeling, and behaviors, especially in relation to the COM-B behavior-change constructs); and other patient, environmental, and contextual factors that may help us understand the trial outcomes and the experience of participants.

The patient perception and experience component of the process evaluation will be conducted at baseline, after enrolment and before a participant is randomized, and at the end of the trial, but before trial outcomes are known. We aim to interview the same participants at baseline and at the end of the trial. Allocation status of participants will be made known to only the process evaluation researchers, with measures to avoid unblinding the trial staff to individual allocation status. We will also conduct two focus groups per site—one male and one female—with a convenience sample of trial participants. Focus groups will be used to achieve a better understanding of experience by allowing a moderated exchange of views among participants. A trained qualitative researcher using a topic guide will carry out the in-depth interviews and focus groups; data will be captured with field notes and through digital voice recording and will be transcribed with anonymization. Notes or transcripts will be coded and themes developed. Standard approaches to ensuring the quality of the methodology will be used, including dual review of transcripts, use of a coding framework, and assessment of samples of dual-coded data.

### Laboratory Measurement

HbA_1c_ was measured using International Federation of Clinical Chemistry calibrated analyzers linked to an international quality assurance scheme at both sites. Total and high-density lipoprotein (HDL) cholesterol was analyzed using an enzymatic colorimetric method, again with an international quality assurance scheme.

### Data Management

Electronic data capture was be carried out using Sana (MIT) utilizing low-cost Android mobile phones and a real-time, mobile Internet connection to a server where the data will be stored using OpenMRS [21]. Final locked versions of deidentified data will be stored in SPSS Statistics for Windows, version 25.0 (IBM Corp). Data records will be available for the 1066 patients recruited to the trial and, we estimate, a further 200 records containing data relating to assessed but ineligible patients. All eligible patients will have two study visits. Data management procedures will follow the detailed data management plan submitted to the funders.

### Quality Assurance Procedures: Bias, Concealment of Allocation, and Attrition

A standardized presentation was delivered to participants emphasizing the importance of lifestyle modification and medicine in treating diabetes. We asked participants not to share the content of their text messages and we did not recruit more than one participant from the same household. Clinic staff do not have access to information about allocated groups and will not have the facility to send text messages to individuals through the study system. Study procedures will be carried out by trained research staff blinded to participant allocation group. Medication dispensing data were collected blind to participant allocation. Study outcomes will be assessed by laboratory staff with no knowledge of treatment allocation or will be assessed by research staff trained not to ask questions that would elicit group allocation.

The study may be monitored or audited in accordance with the current approved protocol—the International Conference on Harmonisation of Technical Requirements for Registration of Pharmaceuticals for Human Use-Good Clinical Practice (ICH GCP)—relevant regulations, and standard operating procedures by the sponsor or funder. The StAR2D Trial Steering Committee and Data Monitoring Committee members are listed in the Multimedia Appendix 3. The sponsor and funder will have no role in interpretation of data or reporting of the trial.

### Publication

The investigators will be involved in reviewing drafts of the manuscripts, abstracts, press releases, and any other publications arising from the study. Authors will acknowledge that the study was funded by the UK Medical Research Council and the Global Alliance for Chronic Disease. Authorship will be determined in accordance with the International Committee of Medical Journal Editors (ICMJE) guidelines and other contributors will be acknowledged.

### Availability of Data and Materials

There are no data relating to this protocol publication; however, following completion, datasets generated during the trial will be available upon reasonable request.

## Results

Recruitment to the trial began in September 2016 and follow-up of participants was completed in October 2018. Data collection from electronic health records and other routinely collected sources is continuing. The database lock is anticipated in June 2019, followed by analysis and disclosing of group allocation. Follow-up of participants at 1 year is complete and data collection from routine sources is continuing. The database has not been locked and trial allocation concealment remains intact.

## Discussion

Type 2 diabetes is a major global health problem, including in sub-Saharan Africa. This is putting additional strain on health services that are often ill-equipped to deal with people with diabetes and other noncommunicable diseases. Thus, there is a need for novel approaches to support health services for people in managing this and other chronic, lifestyle-associated conditions. Digital technology has the potential to deliver interventions at a low cost and at a wide scale. Small effects have been observed in previous work [20,30]. Since this trial was originally funded, further studies have confirmed the potential for using SMS text messaging. A trial among people with type 1 and type 2 diabetes in New Zealand has demonstrated efficacy in reducing HbA_1c_ [31]; in addition, a study using a before-and-after design has demonstrated the feasibility of text messaging in Senegal [32]. However, another study in three countries did not identify a benefit from using SMS text messages in its intervention [33].

This study allows us to explore the impact of SMS text messaging for people with type 2 diabetes in two sub-Saharan sites. The evaluation of effectiveness will provide information about the potential impact of this strategy and its wider cost-effectiveness. The formative work for this trial, along with the process evaluation, will be published separately from the effectiveness and cost-effectiveness evaluations. This study builds on previous work [12], with an increased frequency of messages and a message library utilizing a wide range of behavior-change techniques and an increased number of content domains [18]. SMS text messaging to deliver brief health-related messages is a technology that is already expanding, as access to Internet-based messaging becomes more widely adopted. Nevertheless, the principle of sending brief messages has the potential to be integrated, using a wide range of messaging platforms, into routine care or implemented as *add-on* programs; these could be particularly helpful for between-clinic visit communication and support from health services.

This study focuses on the use of brief messages that could, if necessary, be read by a friend or relative. We have, therefore, not focused on literacy as a potential mediator of impact of the intervention. Future work may need to be carried out to explore the extent to which literacy is needed for this type of brief intervention, although it will clearly be of importance where more extensive use of digital devices is made.

Although this study targets people with type 2 diabetes, the inclusion criteria are wide and do not exclude either participants with comorbid conditions or those with a varied degree of glycemic control. This study provides a further opportunity to explore the potential for delivering care to people with a wider range of cardiometabolic conditions [34], to look at the extent to which the messages meet the different needs of individuals, [35] and the extent to which this technology could be integrated with other aspects of self-management and care delivery.

## Appendix

Multimedia Appendix 1 Participant information sheet.

Multimedia Appendix 2 Template for Intervention Description and Replication (TIDieR) checklist.

Multimedia Appendix 3 SMS-Text Adherence Support for Type 2 Diabetes (StAR2D) Trial Steering Committee and Data Monitoring Committee members.

Multimedia Appendix 4 Peer-reviewer report from the Medical Research Council.

## Abbreviations

COM-B: Capability, Opportunity, Motivation-Behavior
CONSORT: Consolidated Standards of Reporting Trials
DALY: disability-adjusted life year
EQ-5D-3L: EuroQol 5-Dimension 3-Level
HbA_1c_: hemoglobin A_1c_
HDL: high-density lipoprotein
ICH GCP: International Conference on Harmonisation of Technical Requirements for Registration of Pharmaceuticals for Human Use-Good Clinical Practice
ICMJE: International Committee of Medical Journal Editors
ITT: intention-to-treat
NHSRC: National Health Services Research Committee
OpenMRS: Open Medical Records System
OXTREC: University of Oxford Tropical Research Ethics Committee
SMS: short message service
StAR2D: SMS-Text Adherence Support for Type 2 Diabetes
StAR-BP: SMS-Text Adherence Support-Blood Pressure
TIDieR: Template for Intervention Description and Replication
UCT HREC: University of Cape Town Human Research Ethics Committee
