# Supplementary material for: Mobile Messaging Support Versus Usual Care for People With Type 2 Diabetes on Glycemic Control: Protocol for a Multicenter Randomized Controlled Trial
Source: JMIR Res Protoc. 2019 May 30;8(6):e12377. doi: 10.2196/12377 (PMC6592392; doi:10.2196/12377)
Supplement: Multimedia Appendix 1 [file resprot_v8i6e12377_app1.docx]

Thank you for considering participating in the “*SMS supporting treatment for people with type 2 diabetes* “study.

Before you decide to take part you need to understand why this research is being done and what it would involve for you if you took part. Please take the time to read the following information carefully. If you wish to, talk to friends or family about the study as this may help you decide to take part or not. If anything is unclear or you would like to find out more about the study then please contact us. Our contact details can be found at the end of this leaflet.

# Why are we doing this the study?

We are doing this study to find out if using SMS text-messages to keep in contact with people who have sugar diabetes (and who are on treatment) is helpful. We will test whether using different types of message make a difference to blood sugar control.

# Why have I been asked to take part? Can anyone take part?

You are being asked to take part because you are an adult, you have sugar diabetes and you are taking tablets (with or without insulin injections) for your diabetes or are about to start taking tablets (with or without insulin injections) for your high blood sugar. Also, you usually come to this facility for your sugar diabetes treatment.

# What are the other eligibility criteria?

You can choose to take part in this study if:

- You are older than 18 years
- You are taking glucose lowering treatment (pills and or insulin)
- You have a cell-phone (if you share a phone you may take part if the phone owner agrees)
- You know how to use SMS (it is OK if you need help to send or retrieve SMS)
- No one else in your home is already taking part in this study
- You are not pregnant now and have not been pregnant recently (within the past 3 months), also you are not breastfeeding
- You currently live in the community served by the clinic and plan to live here for the next 18 months
- You did not take part in interviews or other work that helped the researchers plan this study

1. **What will happen if I agree to take part?**

- A trained researcher will ask you some basic questions to confirm you are eligible to take part.
- If you choose to take part in the study, one of the research staff will ask you to sign a consent form giving us your written permission to take part. You will be given a copy to keep.
- Once you have signed the informed consent form you will be registered and enrolled into the study.
- We will ask you some questions about any past medical problems.
- We will measure your
  - - height and weight using a study scale
    - blood pressure using an electronic blood pressure device
- We will take a blood sample (5-10ml, about two teaspoons) to measure the levels of HbA1c (sugar) and fats (cholesterol) in your blood at a study laboratory. We will destroy this sample when it has been measured.
- We may also ask you to take part in a group or individual interview about your experiences of taking part in this trial.
- We will send you a SMS text-message to welcome you to the study straight away so you can see what the study SMS text-messages will look like and so you can store the study SMS text-message number in your phone contacts list.
- Please remember we will only send you SMS text-messages from recognized study cell phone numbers. We will never ask for your personal or banking details.

# What will happen after you have received your SMS “Welcome” message?

After today we will stay in contact with you for the next year (12 months) using SMS text-messages. We will send you an SMS text-messages on your birthday and from time to time we will send you other SMS texts-messages about the study.

# Will I have to pay for the SMS text-messages?

No, you will not have to pay to take part in the study. You will not have to pay for receiving SMS text-messages from the study. You will also be able to keep in-touch with the study team at no extra cost.

# Will everyone get the same SMS text-messages?

Everyone in the study will get some SMS text-messages but not everyone will get the same number or type of SMS text-messages. This is because we are looking at the effects of different types of SMS text-messages sent more or less often.

# What should I do with the SMS-text messages I am sent?

The SMS text-messages we send you are for you. If you wish to you can save them on your phone.

# Should I let the study know if I am not getting my SMS text-messages?

The computer system we are using for the study will let us know automatically if you are not receiving the SMS text-messages we’ve been sending you. We will contact you if there is a problem.

# Can I share my SMS-text messages with other people?

Please do not share the SMS text-messages we send you with other people. We are sending the SMS text-messages specifically to you and especially for you.

# How long will the study last?

- The study will last for one year, this means we will stay in contact with you for 12 months from the date of enrolment.
- At the end of the study (in 12-months’ time) we will ask if you would be willing to attend a final follow-up visit for you at a time when it is convenient.
- At the final study visit we will measure your blood pressure, we will re-measure your weight and your waist circumference, and we will take another blood sample (5-10ml, about two teaspoons) to re-measure your blood sugar.
- We will also ask you some questions about how your health was during the year and about whether you found the SMS text-messages helpful.

# What are the possible benefits?

You may find staying in contact with the study via SMS text-message helpful. Information from this study will be helpful to the health services at this and other health care facilities and this could benefit the treatment of patients in the future. If you decide to answer questions about your experiences of taking part in the study you may find this helpful. The information gained from this part of the study will be useful for the researchers and for the health services to learn more about how to design services that use SMS text-messages.

1. **What are the possible risks or discomforts of participating in the study?**

We will ask you to attend two study specific visits (one at the start and one at the end of the study) that may inconvenience you in terms of time. We will use the same equipment and methods to measure your height and weight as in the clinic. We will measure your blood pressure that may cause some slight discomfort. Only suitably qualified persons will take your measurements. We will take two blood samples (one at the start and one at the end of the study) that may cause some discomfort. These procedures carry a small risk of bruising. We will use sterile procedures, and all blood will be taken by qualified and trained individuals to minimise any discomfort. We will not test your blood for any other condition or disease apart from those related to diabetes, and will not use your blood for any other purpose. With your permission, we will pass the results of the sugar and fat tests to your nurse or doctor in the clinic. We will also be collecting your contact details (cellular phone number, address); these will only be available to the research team. The interview questions will not be distressing, but some people may find it emotional or distressing to speak about their experience and their health condition. Interviewers are trained to deal with such situations and to ensure patients are referred for supportive counselling, should this be required.

# What will happen if I don’t want to carry on with the study?

If you decide you do not want to carry on with the study you can contact the study team using a phone, or sending a flash or a “plz cal me”, an SMS text-message or an email (see section 23 for contact details). You can withdraw from the study at any time and you do not have to give a reason. If you decide to withdraw it will not affect your care or treatment at the clinic.

# What will happen at the end of the study?

At the end of this study we will stop sending you SMS text-messages. We will not give your personal information including your blood results to any one without your permission first.

# What will happen to the results of the research?

The research will be published in medical journals. A summary of the results will be presented to all the study participants once this has been done. We will put up a poster explaining the results in the clinic once the study is finished and the results are available.

# Do I have to take part?

No. You may choose if you want to take part in this study. We will explain the study and go through this information sheet with you to answer any questions you may have. If you agree to take part we will ask you to sign a consent form and will give you a copy for you to keep. However, you would still be free to withdraw from the study at any time, without needing to give a reason. If, for whatever reason, you decide to withdraw it will not affect the clinical care you usually receive.

# Will I receive any reimbursements for participating in this study?

We will provide transport to the study site, a meal, and a small stipend to people who participate in this study when they are required to attend a study-specific visit (baseline, final follow-up) at a site other than their regular clinic. We are not providing any phones or airtime credit, all of the study SMS text-messages are provided free-of-charge.

# Who will see the information which is collected during this study?

Your medical records will only be viewed by your health care worker and people involved in this study. Any information collected about you during this study will be kept strictly confidential. Your consent form will be kept in a locked cabinet in a secure building, and will only be able to be accessed by the research team. All other information (your age, whether you are male or female, date of birth, whether you are taking medicines, the measurements made by the blood pressure machines and the blood test results) will not have your name on, and will just have a code so that the results could not be linked back to you. Information provided may be used in publications, reports, web pages, and other research outputs in an anonymised form. Other researchers may have access to this data only in a securely anonymised form. Information you provide may be stored by the project team for a period of time after the project ends. Any personal information about you will be destroyed at the end of the study.

# What if something goes wrong?

The University has arrangements in place to provide for any harm that might arise from participation in the study for which the university is the Research Sponsor. If you have any concerns about any aspect of this study, please contact one of the Clinical Investigators who will do their best to answer your questions. You may contact the UCT Human Research Ethics Committee on 021 406 6338 in case you have any questions regarding your rights and welfare as research subjects on the study.

# Who has reviewed this study?

All research studies are checked by an ethics committee to ensure the research is conducted considering the safety of the participant and to the best standards. This research has been reviewed by and received ethics clearance through the Oxford Tropical Research Ethics Committee and the University of Cape Town Human Research Ethics Committee (OxTREC REF:22-15 and UCT HREC 126/2015).

# Who is organising and funding the research?

This study is being funded by the Medical Research Council and Global Alliance for Chronic Disease.

# What if I have further question?

Please contact one of the local clinical investigators who will happily answer any further questions you may have.

Clinical investigator: Professor N Levitt

Chronic Diseases Initiative in Africa and the Centre of Excellence in Personalized Healthcare

J-floor, Old Main Building Groote Schuur, Cape Town 7937, South Africa

Tel: +27 21 406 6140

Email: liezel.fisher@uct.ac.za

Local Contact: Sr C Delport

Chronic Diseases Initiative in Africa and the Centre of Excellence in Personalized Healthcare

J-floor, Old Main Building Groote Schuur, Cape Town 7937, South Africa

Tel: +27 21 404 2127

Email: cm.delport@uct.ac.za
