# Supplementary material for: Mobile Messaging Support Versus Usual Care for People With Type 2 Diabetes on Glycemic Control: Protocol for a Multicenter Randomized Controlled Trial
Source: JMIR Res Protoc. 2019 May 30;8(6):e12377. doi: 10.2196/12377 (PMC6592392; doi:10.2196/12377)
Supplement: Multimedia Appendix 3 [file resprot_v8i6e12377_app3.docx]

Supplementary Appendix

This appendix has been provided by the authors to give readers additional information about their work.

Supplement to: The StAR2D Trial Collaborative Group. Mobile messaging support versus usual care for people with type 2 in sub-Saharan Africa: a protocol for a multicentre randomized controlled trial (StAR2D). Publication citation to be inserted

**Supplementary Appendix**

**Contents**

Writing Group.............................................................................................................................. 2

Local Investigators....................................................................................................................... 2

Steering Committee....................................................................................................................... 2

Data Monitoring Committee ........................................................................................................... 2

Senior Management Team ............................................................................................................ 2

Coordinating Office (University of Cape Town):............................................................................ 2

Formative and process evaluation ................................................................................................................... 3

Collaborating Centres ................................................................................................................... 3

.....

**Members of Star2D Trial Collaborative Group**

**Writing Group**

A Farmer*, K, Bobrow*, N Leon (NaL), N Williams, E Phiri, H Namadingo, S Cooper, J Prince, L M Crampin, D Besada, E Daviaud, L-M Yu, J N'goma, D Springer, B Pauly, Tarassenko, S Norris, M Nyirenda, N Levitt (NL).

* Equal first author.

Institutions: *Nuffield Department of* *Primary Care Health Sciences, University of Oxford*; AF, L-MY, NW: *Institute of Biomedical Engineering, Oxford*; LT, JP: *The Boston Consulting Group, London, United Kingdom*; DS: *Health Systems Research Unit, South-African Medical Research Council*; NaL, DB, ED: *Cochrane South Africa, South African Medical Research Council;* SC*: Chronic Disease Initiative for Africa, University of Cape Town*; KB, NL: *SAMRC Development Pathways for Health Research Unit, University of Witwatersrand, Johannesburg, South Africa*; SN: *Department of Diabetes and Endocrinology, Chris Hani Baragwanath Academic Hospital, Johannesburg, South Africa*; BP: *Kamuzu Central Hospital, Lilongwe, Malawi;* JN: the *Malawi Epidemiology and Intervention Research Unit, London School of Hygiene and Tropical Medicine, London*: MC, MN: and the *Malawi Epidemiology and Intervention Research Unit, Lilongwe, Malawi*: HN, EP.

.

**Local Investigators**

N Levitt, J N'Goma

**Steering Committee**

*Chair:* M Thorogood; *Chief investigator:* A Farmer; *Statisticians:* L-M Yu, N. Williams; *Administrative coordinators:* S Robinson V Madikizela; *Other members: N* Levitt, P Dorairaj, M Tomlinson, K Bobrow. A Harris representing MRC UK.

**Data Monitoring Committee**

*Chair:* V Cornelius; *Members:* M Bachman, R Mash.

**Senior Management Team**

*Lead:* A Farmer; *Members*: N Levitt, K Bobrow, M Crampin, N Leon.

**Coordinating Office (Chronic Diseases Initiative for Africa, University of Cape Town)**

*Administration and support:* V Madikizela, C Delport, L Fisher (coordinators).

***Statistics and computing support***

University of Oxford: J Prince, D Springer, N Nayan.

**Coordinating Office (Malawi Epidemiology and Intervention Research Unit)**

*Coordinator*: E Piri

**Formative and Process evaluation**

*Lead***:** N Leon**;** *Researchers:* H Namadingo, S Cooper, Namhla Sicwebu

**Collaborators**

*Vanguard Clinic, Bonteheuwel, Cape Town, South Africa*: M Namane*: Kamuzu Central Hospital, Lilongwe, Malawi:* J N'Goma
